# Effects of TMS on Different Stages of Motor and Non-Motor Verb Processing in the Primary Motor Cortex

**DOI:** 10.1371/journal.pone.0004508

**Published:** 2009-02-25

**Authors:** Liuba Papeo, Antonino Vallesi, Alessio Isaja, Raffaella Ida Rumiati

**Affiliations:** 1 Sector of Cognitive Neuroscience, Scuola Internazionale Superiore di Studi Avanzati (SISSA), Trieste, Italy; 2 Rotman Research Institute - Baycrest, Toronto, Ontario, Canada; Università di Parma, Italy

## Abstract

The embodied cognition hypothesis suggests that motor and premotor areas are automatically and necessarily involved in understanding action language, as word conceptual representations are embodied. This transcranial magnetic stimulation (TMS) study explores the role of the left primary motor cortex in action-verb processing. TMS-induced motor-evoked potentials from right-hand muscles were recorded as a measure of M1 activity, while participants were asked either to judge explicitly whether a verb was action-related (semantic task) or to decide on the number of syllables in a verb (syllabic task). TMS was applied in three different experiments at 170, 350 and 500 ms post-stimulus during both tasks to identify when the enhancement of M1 activity occurred during word processing. The delays between stimulus onset and magnetic stimulation were consistent with electrophysiological studies, suggesting that word recognition can be differentiated into early (within 200 ms) and late (within 400 ms) lexical-semantic stages, and post-conceptual stages. Reaction times and accuracy were recorded to measure the extent to which the participants' linguistic performance was affected by the interference of TMS with M1 activity. No enhancement of M1 activity specific for action verbs was found at 170 and 350 ms post-stimulus, when lexical-semantic processes are presumed to occur (Experiments 1–2). When TMS was applied at 500 ms post-stimulus (Experiment 3), processing action verbs, compared with non-action verbs, increased the M1-activity in the semantic task and decreased it in the syllabic task. This effect was specific for hand-action verbs and was not observed for action-verbs related to other body parts. Neither accuracy nor RTs were affected by TMS. These findings suggest that the lexical-semantic processing of action verbs does not automatically activate the M1. This area seems to be rather involved in post-conceptual processing that follows the retrieval of motor representations, its activity being modulated (facilitated or inhibited), in a top-down manner, by the specific demand of the task.

## Introduction

How are individual words represented in the brain and what cognitive operations are required in order to understand them? According to the classical cognitive theories, word representations are abstract and amodal; in other words, they are independent of the sensory and sensorimotor properties of the objects to which they refer (e.g. [1], [2]). Over the last two decades this view has been challenged by evidence to the effect that when people process words related to actions, motor and premotor areas are activated (see [3] for a review), in addition to the *classical* language-processing areas [4]. For instance, Oliveri *et al.* [5] applied transcranial magnetic stimulation (TMS) while their participants performed a morphological transformation task with action-related nouns and verbs and found that activity in the left primary motor cortex (M1) was enhanced regardless of the word's grammatical class. Using a fMRI study, Hauk *et al.* [6] showed that silent reading of action words referring to face-, arm- or leg-related actions, activated areas in the left premotor and primary motor cortices differentially. Tettamanti *et al.* [7] reported somatotopic activation in the left premotor cortex - but not in M1 - during passive listening to sentences implying mouth-, hand- or leg-actions. In both of these imaging studies the motor content of the linguistic material was argued to have automatically recruited the motor programs of the described actions.

These and similar observations have been considered to support the embodied cognition hypotheses of language. Exploiting the Hebbian model of correlational learning [8], Pulvermuller [9] proposed that action words which are typically learned in the context of action performance are represented in the sensorimotor circuits associated with the implied action, woven within the perisylvian-language areas. The *simulationist view* holds that language understanding is achieved via mental simulation of its content by activating traces of previous perceptual and motor experiences in the perceiver's sensorimotor system [10]–[12]. Even though they assume different underlying mechanisms, i.e. associative learning and mental simulation, both proposals predict that understanding action-language *automatically* entails the motor programs of the corresponding physical actions ([3], [11]; see [13] for a review). It follows that a conceptual representation, far from being abstract and symbolic, *is* in fact sensory and motor information.

If action-language processing activates motor representations automatically, then motor activation should occur even when the participants' attention is diverted from the motor content of a word. However, in a recent fMRI study in which task requirements were controlled, M1 activation was only observed in a task in which participants had to imagine the content of motor phrases explicitly, but not in a letter-detection task with the same items [14]. Motor imagery can be triggered even in the absence of explicit instructions, as a strategy to perform any task with sensorimotor components (e.g. [15]–[17]), and it seems to involve M1 particularly when stimuli evoke movements of human body parts (e.g. [18]–[20]). This suggests the possibility that M1 is not an integral part of the network for action-word representation but is recruited only to accomplish tasks that critically require the retrieval of sensorimotor attributes associated with words.

Light can be thrown on the question as to whether motor activation is automatic for action-language understanding by establishing the exact time interval during which M1 activity enhances. Word recognition processing is in fact multistage, characterized by lexical, syntactic, semantic and post-conceptual stages, each with its own specific time course. The hypothesis that M1 is an integral part of word representation implicates that it should be active during the lexical-semantic access, i.e., within 200 ms. Electrophysiological studies seem to support this hypothesis [21]–[23]. Pulvermüller *et al.* [23], using magnetoencephalography (MEG) while participants listened passively to a stream of action words and pseudo-words, reported that a short-lived activity occurred in frontocentral regions within 200 ms after action words appeared. As the technique used has limited spatial resolution, the authors could only conclude that the processing of action words was maintained by “*different parts of frontocentral cortex, possibly including the prefrontal, premotor and motor areas*” ([23], p. 889). On the other hand, in TMS studies, where the temporal resolution is combined with a more precise spatial resolution, language-induced modulation of M1 was found to be either an early phenomenon (e.g., arising when listening to an action verb, before its presentation was over [24]) or a late phenomenon (500 ms post-word, [5]).

The present study addresses two questions. First, does motor activation occur *automatically* even when participants perform a task that barely requires the explicit retrieval of the motor content of the word? Second, which of the different stages of word recognition is most likely to activate the left M1? It was possible to provide answers to these questions using TMS, given the intrinsic characteristics of this technique. In fact, when applied supra-threshold to M1 at a given point in time, TMS elicits MEPs from body muscles as a direct measure of motor excitability at that time: the degree of MEP amplitude following TMS is proportionate to the level of M1 activity. Moreover, when TMS is applied to a brain area it delays or disrupts the ongoing behavior [25]. Therefore the question as to whether M1 activity is necessary for action-language processing can be answered through an investigation of how behavioral performance changes when M1-activity is temporarily altered.

In the present study, TMS was applied to the left hand-M1 and MEPs were recorded from hand muscles while participants performed a semantic task and a syllabic-segmentation task. In the semantic task, participants were instructed to judge whether the presented verbs were action-related, which required explicit retrieval of the representation of the described physical actions. In the syllabic-segmentation task, participants were asked to indicate the number of syllables constituting each verb. The syllabic segmentation primarily entails the sub-lexical features of a word, namely, its orthographical-phonological representation [e.g., 26]. The semantic activation is rather automatic in visual word recognition [e.g.], [27,28], but it might be only implicit when the retrieval of word meaning is not necessary in order to perform the task, as is in the syllabic segmentation. If M1 activation is automatic in response to action words, it follows that when participants perform both syllabic segmentation and semantic encoding tasks, MEPs should be greater for action than for non-action verbs.

Three separate experiments were set up in which TMS was applied at a different point in time after word onset (hereafter, “post-stimulus” will be used synonymously). In Experiment 1, TMS was delivered 170 ms post-stimulus, as the findings of Event-related Potential (ERP) studies indicate that the lexical access for visually-presented words occurs between 100 and 200 ms post-stimulus in posterior regions [29], while there is evidence for early semantic processes starting prior to 200 ms in anterior regions [21], [30]. In Experiment 2, TMS was triggered 350 ms post-stimulus, the time when the brain is thought to encode category-specific attributes of word meaning. In fact, a greater negativity (N400 component) in posterior regions was observed over the 300–350 ms latency range for motor words compared to visual or abstract words [31]. In a similar latency range, differences in parietal and frontal positivity (P300-*like*) correlate with more fine-grained aspects of action-word meaning, such as the body segments involved in the implied action [30]. In Experiment 3, TMS was applied 500 ms post-stimulus, during the post-conceptual stages of word recognition. Manipulating the delay between stimulus onset and magnetic stimulation across experiments served to investigate the time-course of M1 activity when participants performed different linguistic tasks. It also provided a methodological control for distracting/alerting effects and acoustic and tactile sensations associated with TMS. This control is based on the assumption that “non specific effects of TMS will be independent, whereas the behavioral effects will be highly dependent on the precise interval between the event and the stimulation” (see [32] p. 951). This proves to be particularly appropriate for a single-pulse TMS protocol, where stimulus and pulse are not delivered simultaneously [33]. In addition to MEPs, response accuracy and reaction times (RTs) were collected as measures of the participants' linguistic performance. Thus, besides identifying the mental operations that most likely modulate M1 (explicit or implicit encoding of motor content), insight was gained as to *when* M1 is recruited and *what* the nature of its relationship (causal?) is with linguistic performance.

## Results

### Experiment 1: measurement of M1 activity during lexical-semantic access

Eleven right-handed, native Italian speakers participated in this experiment. They were exposed to separate blocks of verbs, selected through a pilot study (see Methods), and were instructed to judge whether they were action or non-action verbs (semantic task) and to indicate the number of syllables (three or other number) composing the verb (syllabic task), through a yes-or-no verbal response. Single-pulse TMS was applied to the left hand-M1 170 ms post-stimulus to elicit MEPs in the first dorsal interosseus (FDI) muscle of the right hand. Each task was composed of two blocks, one using TMS and the other sham stimulation as a control.

Previous TMS studies found an increased hand-M1 activity following rather heterogeneous sets of stimuli, including action verbs related to several body effectors, nouns of manipulable objects [5], or even concrete nouns such as “house” and “collar” [34]. Similar findings have been often explained in the context of an evolutionary scenario whereby language is conceptualized as having evolved from manual communication [34]–[36]. On the other hand, evidence exists that M1 is activated by language in a somatotopic fashion, reflecting the different body-effectors of the implied actions (see [3] for a review). We stimulated the hand-M1, while subjects processed both non action and action verbs. Given the uncertainty about the specific involvement of hand-M1 in language, we considered hand-action and non-hand action verbs as separate levels of the verb-category factor. This yielded a 2×2×3 experimental design with within-subjects factors: (i) stimulation condition (TMS to M1 vs. sham), (ii) task (semantic vs. syllabic), (iii) verb category (hand-action vs. non-hand action vs. non action). Table 1, 2 and 3 summarize the results of the RT, accuracy and MEP analyses, respectively, for the three experiments.

**Table 1:** Mean RTs (ms) in all experimental conditions of Experiments 1–3.

		Semantic task			Syllabic task		
		Hand-act	Non-hand act	Non-act	Hand-act	Non-hand act	Non-act
Experiment 1	TMS	730	726	853	948	905	909
	Sham	722	705	882	818	844	836
Experiment 2	TMS	781	845	**962**	**1156**	**1166**	1105
	Sham	774	848	**886**	**1065**	**1101**	1111
Experiment 3	TMS	627	683	713	843	856	862
	Sham	560	612	683	767	814	762

Tabled mean RTs (ms) following the semantic and the syllabic processing of the hand-action (*Hand-act*), the non-hand action (*Non-hand act*) and the non action (*Non-act*) verbs, during TMS and sham stimulation, in Experiments 1–3. The regions in bold type showed the only significant differences between TMS and sham (Experiment 2).

**Table 2:** Mean Accuracy (proportion of correct responses) in all experimental conditions of Experiments 1–3.

		Semantic task			Syllabic task		
		Hand-act	Non-hand act	Non-act	Hand-act	Non-hand act	Non-act
Experiment 1	TMS	0.93	0.93	0.83	0.87	0.84	0.87
	Sham	0.94	0.91	0.84	0.86	0.86	0.86
Experiment 2	TMS	0.97	0.88	0.88	0.89	0.86	0.88
	Sham	0.99	0.92	0.88	0.93	0.90	0.93
Experiment 3	TMS	0.94	0.90	0.84	0.83	0.84	0.82
	Sham	0.93	0.90	0.90	0.84	0.84	0.82

Tabled mean accuracy (proportion of correct responses) following the semantic and the syllabic processing of the hand-action (*Hand-act*), the non-hand action (*Non-hand act*) and the non action (*Non-act*) verbs, during TMS and sham stimulation, in Experiments 1–3. A difference between TMS and sham stimulation was never observed.

**Table 3:** Means of normalized (sem) MEP peak-to-peak amplitudes in all experimental conditions of Experiments 1–3.

	Semantic task			Syllabic task		
	Hand-act	Non-hand act	Non-act	Hand-act	Non-hand act	Non-act
Experiment 1	−0.05 (0.05)	0.06 (0.08)	−0.02 (0.06)	0.03 (0.07)	−0.09 (0.06)	−0.003 (0.03)
Experiment 2	0.04 (0.05)	−0.17 (0.07)	0.06 (0.04)	−0.04 (0.08)	−0.09 (0.09)	0.06 (0.04)
Experiment 3	**0.12 (0.04)**	0.07 (0.08)	−0.07 (0.04)	**−0.19 (0.07)**	0.01 (0.07)	0.05 (0.05)

Tabled mean normalized MEP amplitude following the semantic and the syllabic processing of the hand-action (*Hand-act*), the non-hand action (*Non-hand act*) and the non action (*Non-act*) verbs in Experiments 1–3. The regions in bold type showed the facilitation in the semantic task and the inhibition in the syllabic task, for hand-action verbs only, as compared to non-action verbs (Experiment 3). A similar dissociation between tasks was not observed for the other verb categories in any of the three experiments.

#### RTs

**Figure 1:**
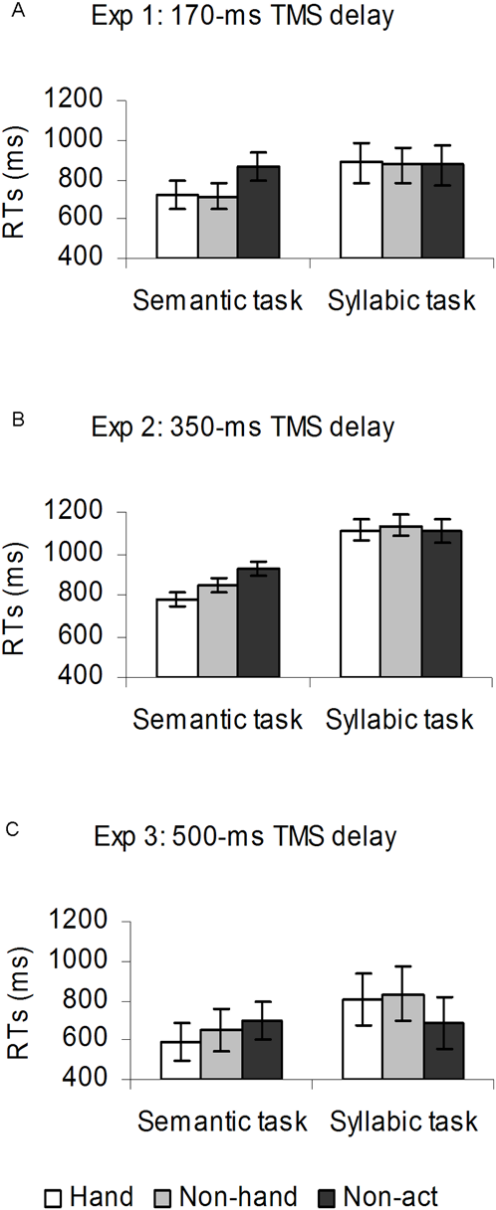
Mean RTs (ms) as a function of the tasks (semantic and syllabic) for the verb categories (hand-action, “*hand*”; non-hand action, “*non-hand*”; and non action, “*non-act*”). Vertical bars denote the Standard Error of the mean. (A) Experiment 1: both the hand-action and the non-hand action verbs were processed faster than the non action verbs in the semantic task; no difference was observed between the verb categories in the syllabic task. (B) Experiment 2: both action-verb categories were processed faster than the non action verbs with an advantage of the hand-action over the non-hand action verbs, in the semantic task. RTs for the three categories did not differ in the syllabic task. (C) Experiment 3: the pattern of performance was consistent with that of Experiments 1–2, although the interaction did not approach significance.

The repeated-measures ANOVA revealed a significant main effect of task *F*(1,10) = 6,27, *p* = 0.03, such as semantic judgments were faster than syllabic judgements (770±72 vs. 876±95 ms; mean±sem). Was also significant the effect of category, *F*(2,20) = 10.33, *p*<0.001, with action verbs (both hand and non-hand related) being processed faster than non action verbs (804±82 and 795±80 vs. 870±88 ms; *p*s<0.001). The task x category interaction was significant, *F*(2,20) = 10.33, *p*<0.001, suggesting that the effect of verb category was dependent on the type of task performed (see Figure 1A). Post-hoc analysis (LSD Fisher's test, α ≤.05) revealed that, in the semantic task, participants responded faster to hand- and non-hand action verbs than non action verbs, (726±69 and 716±70 vs. 867±76 ms; *p*s<0.001), with no difference between the two action-verb categories (*p*>0.1). Instead, the three verb categories did not differ in the syllabic task (884±96 and 875±90 vs. 872±99 ms; *p*s>1). This effect was independent of TMS, as the interaction between stimulation condition, task and category did not approach significance, *F*(2,20)<1, n.s. Thus, the semantic encoding was faster for the action than for the non action verbs. This difference disappeared when performing the task did not rely on the word meaning, as in the syllabic segmentation.

#### Accuracy

**Figure 2:**
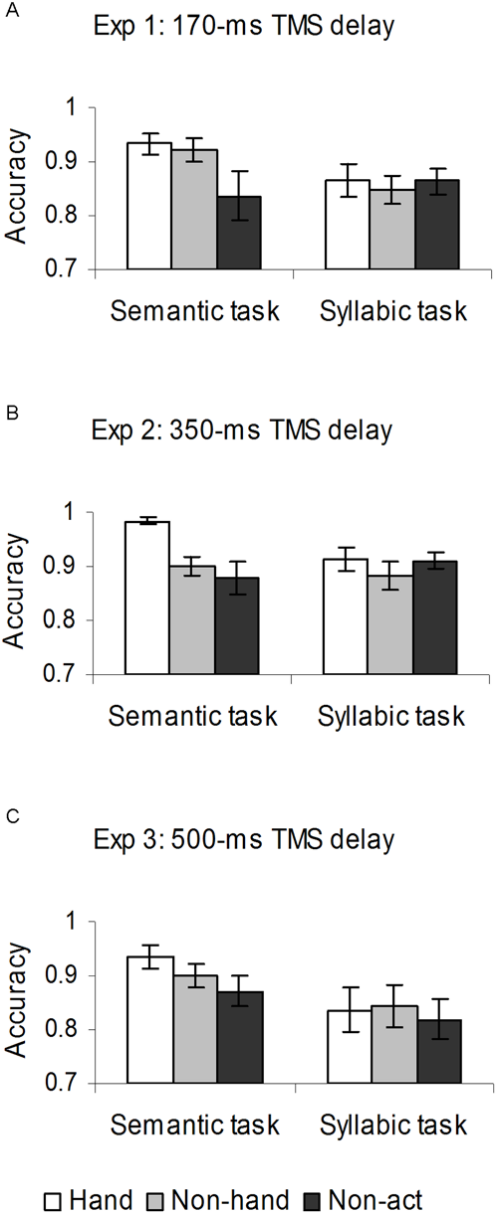
Experiment 2: Mean accuracy (proportion of correct responses) as a function of the tasks (semantic and syllabic) for the verb categories (hand-action, “*hand*”; non-hand action, “*non-hand*”; and non action, “*non-act*”). Vertical bars denote the Standard Error of the mean. (A) Experiment 1: in the semantic task, both the hand-action and the non-hand action verbs were processed more accurately than the non action verbs; no difference was observed between the verb categories in the syllabic task. (B) Experiment 2: hand-action verbs were processed more accurately than the other verb categories in the semantic task; the three categories did not differ in the syllabic task. (C) Experiment 3: descriptively, the pattern of performance was consistent with that of Experiments 1–2, although the interaction did not approach significance.

The effect of task resulted significant, *F*(1,10) = 5.13, *p*<0.05, semantic judgments being more accurate than syllabic judgments (0.90±0.03 vs. 0.86±0.03, mean proportion of correct responses±sem). There was a trend for the interaction between task and category, *F*(2,20) = 2.77, *p* = 0.08 (see Figure 2A). Post-hoc comparisons showed that the semantic judgments were more accurate on the two categories of action verbs, relative to non action verbs (0.93±0.02 and 0.92±0.02 vs. 0.83±0.04; *p*s<0.05), whereas the syllabic task was performed equally well with the three categories (0.86±0.03 and 0.84±0.03 vs. 0.86±0.02, *p*s>0.6). This pattern was consistent with the RT results, and allows us to rule out the speed-accuracy trade-off as an explanation for the observed performance.

#### MEPs

MEPs (mV) recorded from the right FDI muscle during TMS delivery, were normalized. Mean z-scores of MEP peak-to-peak amplitude were subjected to a 2×3 repeated-measures ANOVA with task and category as factors. No effect or interaction approached significance (all *p*s>0.2). Although action verbs enjoyed a temporal advantage on non action verbs, in semantic processing, the resulting early lexical-semantic access did not elicit a specific enhancement of M1 activity.

### Experiment 2: measuring M1 activity during semantic-attribute processing

A total of 14 right-handed, native Italian speakers took part in Experiment 2. The experimental design and statistical analysis were identical to those used in Experiment 1, with the sole difference that, here, TMS was applied 350 ms post-stimulus.

#### RTs

The effect of task was significant, *F*(1, 13) = 13.53, *p* = 0.003, as was the effect of category, *F*(2,26) = 12.00, *p* = 0.001. RTs in the syllabic task were slower than those in the semantic task (1117±104 vs. 849±66 ms), and hand-action verbs were processed faster than non-hand action verbs and non action verbs (944±82 vs. 989±83 and 1016±89 ms; *p*s<0.01). The task x category interaction resulted significant, *F*(2, 26) = 10.4, *p*<0.01 (see Figure 1B). In the semantic task, hand-action and non-hand action verbs were processed faster than non action verbs (778±61 and 846±63 vs. 924±73; *p*s<0.01). The difference between the two action-verb categories was also significant, with hand-verbs being judged faster than non-hand action verbs (*p*<0.01). The three categories did not differ in the syllabic task (1111±105 and 1133±104 vs. 1108±105; *p*s>0.2). A significant TMS x task x category interaction was also found, *F*(2, 26) = 4,27 *p* = 0.02 (Table 1). In the semantic task, TMS further delayed the participants' performance on non action verbs compared with the sham condition (*p* = 0.02). No effect of TMS was observed in the semantic task for the two action-verb categories (*p*s>0.1). Conversely, in the syllabic task TMS slowed down responses to both action-verb categories compared with the sham condition (*p<*0.05). As for Experiment 1, participants processed action verbs faster than non-action verbs in the semantic but not in the syllabic task. In addition, the three-way interaction revealed that TMS delivery to M1 inhibited participants' responses when they performed the semantic task with non action verbs, and when they performed the syllabic task with action verbs.

#### Accuracy

The ANOVA revealed a significant main effect of TMS, *F*(1, 13) = 6,83, *p* = 0.02, with participants being less accurate during TMS than sham stimulation (0.90±0.02 vs. 0.92±0.02) . However, this factor did not interact with any other factor in the design. The main effect of category was also significant, *F*(2, 26) = 10,59, *p*<0.001, such as processing hand-action verbs was more accurate than processing hand-action and non-action verbs (0.95±0.01 vs. 0.89±0.02 and 0.89±0.02; *p*s<0.001). The ANOVA also indicated a significant task x category interaction, *F*(2, 26) = 4,71, *p* = 0.02 (see Figure 2B). Particularly, participants performed semantic judgements more accurately on hand-action verbs than on non-hand action and non-action verbs (0.98±0.007 vs. 0.90±0.02 and 0.88±0.03; *p*s = 0.001). No difference was observed between action and non-action verbs in the syllabic task (0.91±0.02 vs. 0.88±0.02 and 0.90±0.02; *p*s>0.1). This ruled out the speed-accuracy trade-off effect as an explanation for the RT results, confirming that the semantic task was more difficult when non action verbs were involved, and that this difference disappeared when processing phonological aspects of verbs.

#### MEPs

The ANOVA of mean MEP peak-to-peak amplitudes revealed only a trend for the effect of category, *F*(2, 26) = 3,26, *p* = 0.05. The MEP amplitude was the greatest for non-action verbs. However, the lack of interaction between task and category, [*F*(2, 26)<1, n.s.] did not support any obvious conclusion regarding a specific involvement of left M1 in word processing.

### Experiment 3: measuring M1 activity during post-conceptual processing

Experiment 3 involved 11 new participants. The procedures were identical to that of Experiments 1–2, the sole difference being that the delay between stimulus onset and TMS delivery was 500 ms.

#### RTs

The RT analysis revealed a significant main effect of task, *F*(1, 10) = 13.95, *p*<0.01, with the semantic task being faster than the syllabic (646±103 vs. 817±135 ms). The effect of category was also significant *F*(2, 20) = 6.03, *p*<0.01. Again, hand-action verbs were processed faster than non-hand and non action verbs (699±115 vs. 742±125 and 755±116 ms; *p*s<0.05). Basically the pattern of the interaction between task and category was comparable to Experiments 1–2 (see Figure 1C), although it did not approached significance, *F*(2,20)<1, n.s.

#### Accuracy

The ANOVA revealed a significant main effect of task, *F*(1,10) = 5,27, *p* = 0.04: the semantic task was performed better than the syllabic task (0.90±0.02 vs. 0.83±0.004). Descriptively, the pattern of participant's performance in the two tasks, with the three verb-categories was consistent to Experiments 1–2 (see Figure 2C), but no interaction approached significance (all *p*s>0.1).

#### MEPs

The ANOVA showed a significant task x category interaction, *F*(1, 10) = 8.872, *p* = 0.01. Post-hoc analyses showed that the semantic processing of hand-action verbs elicited greater motor activation compared with non action verbs (*p* = 0.03), whereas the MEP amplitude for non-hand action did not differ from that for non-action verbs (*p*>0.1). In the syllabic task, the difference in the level of M1 excitability after processing hand-action verbs was significantly smaller relative to non-hand action and non action verbs (*p*s*<*0.03). Again, there was no difference in the MEP amplitude between non-hand action and non action verbs (*p* = 0.6). A difference was observed in the MEP amplitude between the semantic and syllabic processing of hand-action verbs (*p* = 0.001): M1 activity significantly increased and decreased depending on whether the same verbs were processed semantically and syllabically, respectively. A similar difference between tasks was not observed for the other two categories (*p*s>0.1). Thus, the enhancement of M1 activity occurred only when participants explicitly encoded the content of the hand-action verbs, but not when they encoded their phonology. In the latter condition, M1 activity resulted to be rather inhibited. Mean normalized MEP amplitudes for all the conditions of Experiments 1–3 are listed in Table 3.

### Between-subjects analysis

**Figure 3:**
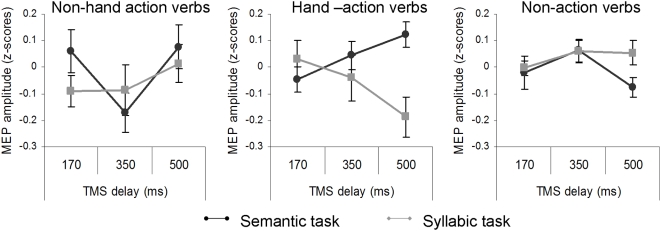
Analysis of normalized MEP amplitude for the verb categories (hand-action, non-hand action and non action verbs) as a function of the tasks (semantic and syllabic) and the timing of TMS delivery (170, 350, 500 ms) as the only between-subjects factor. At 500 ms post-stimulus, MEP amplitude increased when the participants performed the semantic task with hand-action verbs compared with non-action verbs. It decreased, relative to non action, when the participants performed the syllabic task with the same hand-action verbs. A similar dissociation between M1 activity associated with the two task conditions was never observed for the non-hand action verbs. Vertical bars denote the Standard Error of the mean.

MEP data from all the three experiments were subjected to an ANOVA with factors, 2 task and 3 category manipulated within subjects, and 3 timing of TMS delivery as a between-subjects factor. This analysis was performed in order to investigate the time-course of M1 activity associated with each verb category during their semantic and syllabic processing. The three-way interaction between task, category and TMS timing approached significance, *F*(4,66) = 2,1656, *p* = 0.08 (see Figure 3). Post-hoc comparisons revealed a different pattern of M1-activity for hand- vs. non-hand action verbs, when compared with non action verbs.

#### Hand-action verbs

At the first two timings of TMS delivery (i.e., 170 and 350 ms post-stimulus), MEP amplitude for hand-action verbs was not different from that for non action verbs, in either task (all *p*s>0.3). Moreover, at these latencies, MEP amplitude following the semantic and the syllabic processing of hand-action verbs did not differ (*p*>0.3). When recorded at 500 ms post-stimulus, the difference between MEPs for hand-action verbs and non-action verbs approached significance in the semantic task (*p* = 0.07), and reached significance in the syllabic task (*p* = 0.03). Moreover, M1 activity for hand-action verbs resulted greater in the semantic than in the syllabic task (*p*<0.01). Confirming those from the individual experiments, these findings suggest that M1 activity is modulated by hand-action verb processing only during post-conceptual stages of word recognition (500 ms post-stimulus), with the direction of the modulation (increase or decrease) depending on the task-demand.

#### Non-hand action verbs

The pattern of M1-activity following non-hand action verbs proved to be different from that of hand-action verbs. The MEP amplitude associated with this verb category did not differ from that of non-action verbs in either task condition, and at any time interval (all *p*s>0.2), except at 350 ms. At this latency, the MEP amplitude decreased for non-hand action verbs relative to non action verbs in the semantic task only (*p* = 0.02). In the same condition, it was significantly smaller even when compared with the MEP amplitude associated with hand-action verbs (*p* = 0.03). A difference between the two action-verb categories was also observed at 500 ms: here, MEP amplitude for non-hand action was greater than that for hand-action verbs in the syllabic task (*p* = 0.02). This pattern suggests that the semantic and the syllabic processing of the non-hand action verbs did not elicit motor facilitation and inhibition, respectively, when compared with non-action verbs. No difference was observed in MEP amplitude when the same non-hand action verbs were subjected to the two tasks (*p* = 0.5), so that the dissociation in MEP amplitude between semantic and syllabic tasks, observed for hand-action verbs at 500 ms post-stimulus, never occurred for non-hand action verbs.

The results from the between-subject analysis confirmed the findings from the single experiments. The effect of action-verb processing on M1 activity was observed at 500 ms post-stimulus when the hand-M1 activity following hand-action verbs increased during the semantic task, and decreased during the syllabic task. Motor activation associated with action verbs did not occur within 350 ms, i.e. in the time interval for lexical-semantic access. We suggest that the phenomenon of language-induced motor resonance in the hand-M1 took place only after the lexical-semantic access to representations of hand-action verbs, and only when the task explicitly required a full processing of the motor information associated with a word. At the same latency, the syllabic segmentation of the same hand-related items led to an inhibition of the motor activity. The fact that this pattern was observed only with hand-action verbs, indicates that the phenomenon of motor resonance can actually occur in a somatotopic fashion, reflecting the implied-language content.

We cannot draw any definitive conclusion about the processing of non-hand action verbs. It is possible that different M1-sites, other than the hand-M1 area, were activated when participants processed non-hand action verbs. Since we only stimulated the hand-M1 area, we cannot exclude that any effect we observed when participants processed non-hand action verbs, was due to the activation of motor-sites, other than the hand-M1, spreading through the horizontal cortico-cortical connections [37]. On the other hand, the differential effect on hand-action and non-hand action verb processing when TMS was delivered to hand-M1, suggests that the interaction between action-word meaning and motor system is sensitive to the somatotopic organization of M1, as documented by previous studies [e.g], [24, 38].

## Discussion

This study challenges the view that action-language processing automatically activates motor representations in the brain. TMS was used to measure the excitability of the participants' left primary motor cortex while they processed action verbs. In order to establish whether M1 activity is causal to language processing, RTs and accuracy were analysed to assess whether action verb processing was modified as an effect of the TMS-interference with this region.

We found that participants were faster (and better) at processing action verbs (both hand and non-hand related) as opposed to non-action verbs, when they had to access their semantic content but not when a syllable count was required (Experiments 1–2). Relative to sham, TMS delayed the semantic judgments of non-action verbs and the syllabic segmentation of action verbs when applied 350 ms post-stimulus (Experiment 2). Although the semantic processing was sometimes faster and more accurate for the hand-action than the non-hand action verbs (Experiments 2–3), these two categories interacted similarly with the other factors (i.e., task, in Experiment 1; task and TMS condition, in Experiment 2), and differently with respect to non-action verbs, suggesting that they can be considered as a unique lexical-semantic category. No enhancement of the hand-M1 activity emerged within the response latency for lexical-semantic word processing (Experiments 1–2). The hand-M1 activity was modulated by language only when measured at 500 ms post-stimulus. Specifically, relative to non-action verbs, M1 activity increased when participants performed the semantic task with hand-action verbs, and decreased when they performed the syllabic task with the same items (Experiment 3). A similar pattern was not observed for non-hand action verbs: these stimuli elicited the same activation of M1 as the non-action verbs, both in the semantic and in the syllabic task. The MEP analysis with the timing of TMS delivery as a between-subjects factor fully confirmed the findings of the three experiments.

The overall advantage of action verbs relative to non-action verbs is established by literature on lexical-semantic processing. Kellenbach *et al.* [31], using ERP, found that the earliest effect associated with semantic attributes was elicited by motor words at about 250 ms, compared with abstract and visual words. However, while this study revealed a temporal precedence of the cortical response to motor attributes (versus non-motor attributes), it did not find distinct cortical networks involved in processing the different word categories [31]. This is consistent with the behavioral findings of Laws *et al.* [39], who showed that healthy participants were faster at performing a sentence-verification task with associative-functional (i.e. motor) attributes as opposed to visual attributes, possibly because the former category is characterized by a more extensive and complex cortical representation than the latter. In fact, the motor information “attached” to action-word representations is held to provides a relational context - including path, manner, results and instruments [40] - that enriches and instantiates their conceptualization. Hence, action verbs are more concrete than non-action verbs and evoke mental images more strongly. These two dimensions (i.e. *concreteness* and *imageability;* [41]) are known to affect word retrieval and recognition in both healthy and brain-damaged individuals [42], [43]: the more concrete and imaginable the word is, the faster it will be processed.

In Experiment 2 two further effects were observed: compared with the sham condition, the application of TMS to M1 delayed the RTs of (1) the semantic encoding of non-action verbs, and (2) the syllabic segmentation of action-verbs (both hand and non-hand related). The first effect can be the consequence of accessing the semantics of words with *non-motor* content while a concurrent motor stimulation was provided. In fact, by eliciting overt hand movements, TMS might have acted as an incongruent prime-*like* stimulus when participants processed non-action word content (see also [38]). Probably, the lack of facilitation in the semantic processing of action verbs is due to the fact that this was the fastest experimental condition, so that RTs could not be further shortened - with a statistically significant time period - by a congruent stimulation. The second effect is possibly explained by a similar mechanism. In fact, although the syllabic segmentation primarily involved phonological operations, an automatic semantic access occurs whenever a word is read [27], [28]. It is also known that when multisensory stimuli co-occur, the information they convey is combined to generate a response [44]. Thus, a concurrent motor stimulation (or the view of hand movement elicited by TMS) during the processing of action verb, would facilitate the *automatic* activation [28] of the motor information associated with the word. This information, being irrelevant to the task, might have been inhibited before responding, with a processing cost reflected in RTs [45]–[47]. Critically, these effects were observed just at the latency (350 ms) when word's motor or non-motor attributes are supposed to be processed, and were not accompanied by a specific M1 activation, as demonstrated by the MEP analysis. Since the *effects of congruence* are known to occur when stimulus and response or two concurrent stimuli overlap for any dimension [48], in the context of our study, they suggest that language and motor systems actually interact. However, this interaction seems to occur at the stage of processing when abstract, conceptual representations of words and actions are contacted, and does not recruit lower-level motor programs, contrarily to what suggested by the embodied models [10]–[13]. Moreover, the interaction with the hand-stimulation was similar for hand-action and non-hand action verbs, as if a more symbolic and abstract representation of “motion” associated with the word, rather than a specific motor program, was involved. A similar interaction between M1 stimulation and word meaning even if with earlier delay (i.e., 150 ms), has been reported by Pulvermüller *et al.* [38], even with methodological differences (i.e., time and intensity of TMS and task demand) relative to our study. In their study, TMS-pulse acted as a semantic prime-stimulus that facilitated (i.e., faster RTs) the lexical decision on hand-words when applied to the hand-M1, and on leg-words when applied to the leg-M1. No measure of M1 activity was available as TMS here was applied sub-threshold. By inducing a priming effect with TMS, this study provides a very elegant evidence of the interaction between language processing and M1 activity. In our study, behavioral and MEP results demonstrated that this interaction can occur even in the absence of a motor activation specific for action language.

M1 activity increased when TMS was applied 500 ms post-stimulus, only when participants performed the semantic task with hand-action verbs, suggesting that language-induced motor activation depends upon the *explicit retrieval* of the action content of the word. Conversely, in the syllabic task, we observed a decrease of M1 activity following hand-action verbs, possibly reflecting an inhibition of the motor processes that were not required by the task. The increase and the decrease of corticospinal excitability have been previously associated with the facilitation and the inhibition of motor processes respectively [49], [50]. In particular, in Koch *et al*.'s [49] study on functional connectivity of premotor and motor areas, MEP amplitude increased when a response had to be performed, and decreased when a response was one of the possible alternatives but not the correct one, and therefore, had to be suppressed. Interestingly, M1 activity was modulated, in a top-down manner, by higher-level motor areas (i.e. the premotor cortex) *choosing* between alternative responses for satisfying the task demand. Similarly, in our study, the modulation of hand-M1 activity (increase or decrease) resulted to be constrained by whether the task required a full processing of the motor content of the word, that in turn would elicit the motor simulation, or not. The specificity of the effect for hand-action verbs, when only hand-M1 was stimulated, is consistent with the hypothesis that language-induced motor resonance can be actually explained by imagery processes [14]. In fact, evidence exists that M1 is involved in motor imagery in a somatotopic fashion (e.g. [18]) and the present findings add that, during language processing, this is accomplished strategically rather than an automatically. The late *co*-occurrence of M1 activation, together with the absence of relationship between this activation and the linguistic performance (RTs and accuracy), critically contribute to this conclusion, against the view that the phenomenon of language-induced motor activity automatically occurs during the lexical and semantic stages of word recognition, and is causal to them.

Although electrophysiological studies in which participants were exposed to action-language, provided evidence in support of frontocentral activation occurring within 200 ms [21], [23], the limited spatial resolution of these techniques did not allow to locate with precision the site of the activations in motor and premotor regions. On the other hand, TMS studies did reveal an involvement of M1 during action-language processing, but left the time and the direction (increase or decrease) of the effect unclear. In fact, consistently with our findings, Oliveri *et al.* [5] observed an increased M1 activity 500 ms post-stimulus, while participants produced action words in a transformation task. In contrast, Buccino *et al.* [24] found that listening to hand-sentences decreased hand-M1 activity and listening to foot-sentences decreased foot-M1 activity. These effects occurred quite early after the onset of the critical action verbs (TMS was indeed delivered when the second syllable of the verb was presented). While this study provide a clear evidence for a specific modulation of action language on M1 activity, the use of a passive task and, therefore, the lack of an online measure of the participants' performance, make it difficult to establish what sort of processing they have been actually performing, when M1 was stimulated. It thus seems premature to conclude, from these results, that M1 is *causally* involved, or what its functional relevance is, in action language processing (but see [51] for a possible interpretation of conflicting results). Moreover, none of the abovementioned studies tested M1 activity using the same stimuli under different task conditions and at different times during word processing. The latter manipulation was carried out in a TMS study by Meister *et al.,* [34]. They measured the left-M1 excitability at different time intervals while participants were reading concrete nouns aloud, and found enhancement only at 600 ms post-stimulus and later. To the best of our knowledge, this time interval is far beyond that for lexical-semantic access and might rather reflect post-conceptual processes elicited by concrete, highly-imaginable items. The existence of post-conceptual stages of language processing has been suggested by the activation of a fronto-temporal network, after the lexical-semantic access, that would sustain a supramodal-contextual integration of different levels of information from different modalities [52]. In the case of action verbs, the retrieval of conceptual representations would lead to the implicit generation of the mental images associated with them that, in turn, activate M1 (e.g. [17]–[19]). It is known that this motor imagery is automatically engaged as a strategy to perform tasks with sensorimotor components (here the semantic encoding of action verbs) that “cannot easily be inferred from […] verbal material” ([53], p. 1337) or, we add, whose recall is more effective using motor simulation. Interestingly, Tomasino *et al*. [54] have shown that sub-threshold TMS to M1, acting as a prime stimulus, facilitated responses in an imagery task with action verbs (judging whether they described hand-rotation) but not in reading and frequency judgments of the same items. This effect extended beyond 750 ms post-stimulus, which is consistent with the lengthy time interval typically associated with motor imagery [55]. Analogously, the time at which language-related M1 activation (500 ms) was observed in the present study, fell fairly within the interval for imagery processes (from 400 ms to 750 or more; [20], [56]). It is fair noting that, in Tomasino *et al*., the effect began at 150 ms post-stimulus, whereas in our study it was observed only later (after 350 ms). This timing discrepancy can be clearly related to the different tasks employed in the two studies. In Tomasino *et al*., participants were explicitly required to imagine the content of the verbs, so that imagery presumably *started* as soon as the word appeared. In our paradigm, imagery was not explicitly required, and it only kicked in language processing when it proved to be an effective strategy to solve the task.

We propose that M1 activation would *result from* understanding action verbs rather than *contributing to* it. Studies carried out with critical populations have already suggested that action comprehension can be achieved without mental simulation. Infants and children were able to recognize meaningful actions even when motor simulation was not available because the actions violated the human-body constraints (i.e., impossible actions, [57]), or did not belong to the human motor repertoire (i.e., the agents were non-human, [58]). In addition, double dissociations between action performance and action-language processing have been documented in neuropsychological investigations, suggesting that language *is still possible* with disrupted motor representations and vice versa [59], [60].

This study demonstrates that the motor activation related to action language is not strictly necessary to its understanding in a narrow sense (i.e., lexical-semantic encoding). This phenomenon is more likely to reflect post-conceptual operations resulting from the explicit retrieval of the motor information associated with action language, when this is critical to solve a task. Future research will clarify the functional relevance of this post-conceptual operation for language understanding. Here, we suggest that the strategic function of motor imagery can provides an insight as to why language processing goes beyond the completion of lexical-semantic encoding and engages the motor system.

## Materials and Methods

### Participants

Eleven individuals (7 men, 4 women; mean age  =  24.5±4 years) took part in Experiment 1, 14 (5 men, 9 women; mean age  =  25.7±3.5 years) in Experiment 2, and 11 (5 men, 6 women; mean age  =  26.3±5 years) in Experiment 3. All the participants were right-handed (mean laterality quotient in Experiment 1: 83, range 65–100; in Experiment 2: 80, range 60–100; in Experiment 3: 86, range 65–100; [61]), and had normal or corrected-to-normal vision. None of them had ever participated in a TMS experiment before. The participants were provided with an explanatory leaflet on TMS prior to the experiment, and filled in a questionnaire to ensure they were clear of contraindications to TMS [62]. They confirmed their voluntary participation in writing, gave their written consent, and received compensation for their collaboration. The study was approved by the local ethics committee.

### Stimuli

Before proceeding to the actual experiments, a pilot test was conducted, in which a list of 375 verbs was shown to ten participants who were not involved in the main experiments. This set of items was selected following the criteria of the linguistic tradition, whereby action verbs refer to physical acts, and non action verbs (state/psychological verbs) expressed mental processes with no reference to a physical object [63], [64]. Participants were asked to decide whether the verbs were action-related, and for those that were, to specify the associated body effector among the following alternatives: “upper limb”, “lower limb”, “head” or the “whole body”. The verbs designated as action-related by at least 80% of the panel were included in the TMS study, resulting in a final set of 256 items. This set included 128 action-verbs (64 associated with hand motion, e.g. *“mescolo”*, *I stir;* and 64 associated with other body effectors, *“salto”, e.g., I jump*) and 128 non-action verbs (e.g. “*medito*”, *I wonder*), all in the first person singular of the present tense (see Appendix S1). Fifty percent of the hand-action, the non-hand action and the non action verbs were 3-syllable words; the other fifty percent was divided equally between 2- and 4-syllable words. In addition to length (i.e., number of syllables), hand-action, non-hand action and non action verbs were matched for written frequency (Dizionario di frequenza della lingua italiana, Consiglio Nazionale delle Ricerche, C.N.R.- I.L.C.), *t-tests* n.s.

### Procedure

Participants performed two tasks, a semantic and a syllabic segmentation task, both of which involved action and non-action verbs. In the semantic task, they were asked to judge explicitly whether the verb implied a physical act (e.g. *“mastico”*, *I chew*) or a psychological or mental state (e.g. *“adoro”*, *I adore*). In the syllabic-segmentation task the participants indicated the number of syllables of each verb (3 or different from 3, i.e., 2 or 4). They sat on a height-adjustable chair at approximately 1 meter from a 17′ CRT screen that displayed the stimuli (font: Arial 38). The height of the chair was regulated to align the participants' gaze with the centre of the display. Each trial began with an acoustic alert of 1500-Hz pure tone followed by a blank screen for 100 ms, which was followed in turn by a fixation cross displayed in the centre of the screen for 1750 ms. A 200-ms blank then appeared. Afterward, the verb was projected in the centre of the screen for 375 ms, which gave the participants sufficient time to read the stimulus [29]. The verb was then substituted by three dots which were projected for 3975 ms, to allow participants to provide the vocal response. On conclusion of this cycle, the next trial began. Each trial lasted 6200 ms from start to finish, sufficiently long to prevent interaction between consecutive TMS-pulses [32]. The participants were instructed to give yes-or-no vocal responses to all the stimuli in both tasks. Half of the participants had to give the yes-response to action-related verbs and the no-response to non action verbs in the semantic task, while in the syllabic task the yes-response was to correspond to 3-syllable verbs and the no-response to 2- or 4-syllable verbs. The other half of the participants received opposite instructions. The voice-onset time was recorded as a measure of RTs, using a microphone connected to the external response box of an E-prime PC-controlled system (Psychology Software Tools, Inc., Pittsburgh, PA). Response accuracy was recorded by the experimenter who pressed one of the two mouse keys: the right for yes-responses and the left for no-responses.

The experiment consisted of four blocks of 64 trials each, for a total of 256 items: a semantic task (64 verbs) and a syllabic task (64 verbs) with TMS, and a semantic task (64 verbs) and a syllabic task (64 verbs) during the sham stimulation. 128 MEPs were obtained for each participant, one magnetic stimulus being applied for each item (the pulses delivered during the two sham-blocks did not elicit MEPs). Action and non-action verbs were presented in a random order within each block, with a short pause after 32 items. Participants were given four practice trials before each block. The order of the two tasks, the mapping of the verb type (action vs. non-action verb and 3 syllables vs. 2 and 4 syllables) to a response (“yes” or “no”), and the verb lists in TMS and the sham condition were all counterbalanced across participants.

### Single-pulse TMS protocol

#### TMS site and TMS intensity

Single-pulse TMS was applied to the left M1, using a Magstim 200 stimulator (Magstim Company, Withland, UK) connected to a figure-of-eight coil (70 mm in diameter). The coil was positioned by mapping the cortical representation of the first dorsal interosseus muscle (FDI) of the right hand, starting from the Cz reference point of the international 10–20 EEG system [65] and moving the center of the coil approximately 6 cm to left, i.e. position C3/C4. The optimal scalp position for the induction of MEPs with the maximum amplitude in the right FDI muscle was individuated for each participant. The coil rested tangential to the scalp surface. The target site was marked on the participant's head with a cosmetic pencil, and the coil was maintained in position by an articulated, metallic arm.

The TMS intensity was adjusted to 120% of the motor threshold at rest, which is defined as the minimum intensity to evoke MEPs with≥50 µV peak-to-peak amplitude in the relaxed FDI, in 3 out of 5 consecutive pulses [66]. The mean motor threshold for participants of Experiment 1 was 37.6±1.4% of the maximum stimulator output. The means in Experiment 2 and 3 were 38.7±1.4 and 38.9±2.2 respectively. Participants were instructed to keep their right arm/hand and head motionless and the muscle relaxation was monitored throughout the entire experiment to check for involuntary movements. A visual feedback consisting of a muscle twitch, i.e. an abduction movement of the right forefinger, was always present after actual TMS delivery.

The same intensity of magnetic pulses was used for both the TMS (2 blocks) and the sham stimulation (2 blocks). In the latter condition, the coil was held perpendicularly to the surface of the scalp over the left M1, so that it mimicked the noise and the mechanical vibration of TMS but no magnetic stimulation actually reached the scalp [32]. The order of the two stimulation conditions was counterbalanced across subjects according to Latin square. Participants were not informed whether they were going to receive TMS or sham stimulation. The sham stimulation was used as a control for the diverting effects of the acoustic and tactile sensations associated with TMS. However, the intensity of these peripheral effects is typically lower in the sham condition than in TMS and only TMS elicits muscle twitches and stimulates facial muscles. To reduce the possibility of participants to make an a priori distinction between the two conditions, we selected only participants who had been never exposed to TMS before. In fact, after the experiment, when we debriefed them, they all reported the sensation that “something was going on”, even during the sham condition. This was only a very general precaution for preventing the effect of awareness from crucially affecting the performance. More to the point, the nonspecific effects of TMS were controlled by manipulating the timing of TMS delivery in the three different experiments, and by performing the between-subjects analysis of the MEP data.

The same protocol was adopted for all three experiments, the only difference being the timing of the TMS application. In Experiment 1, TMS pulses were triggered 170 ms after the onset of each stimulus. In Experiment 2, TMS was applied 350 ms post-stimulus and 500 ms post-stimulus in Experiment 3. TMS-induced MEPs were recorded by a pair of gold surface electrodes placed over the FDI (active electrode) and the metacarpophalangeal joint of the index finger (reference electrode). The ground electrode was placed on the ventral surface of the right wrist. The electromyographic (EMG) signal was amplified and filtered (bandpass 20 to 2000 Hz) through a Grass amplifier (P122 Series) and recorded with the Biopac system (MP150 model) at a sampling rate of 5 kHz. EMG data were transferred to a personal computer for offline analyses of the MEPs using Matlab (The MathWorks, Natick, Massachusetts, USA). The delay of TMS delivery in each experiment was replicated in the sham condition.

### Analysis

The same statistical analyses on RT, accuracy and MEP data were performed in Experiments 1–3 as the experimental design itself was identical. Practice trials, trials with RTs shorter than 100 ms and longer than 2500 ms, and those in which the participants made errors in the syllable count or in semantic judgement (according to the pilot study), were excluded from the offline analysis. Mean RTs and accuracy were submitted to a 2×2×3 analysis of variance (ANOVA), with TMS condition (left M1 vs. Sham), task (semantic vs. syllabic) and verb category (hand-action vs. non-hand action vs. non action) as within-subjects factors.

In the MEP analysis the peak-to-peak amplitude (mV) of each MEP was computed by an automatic Matlab script and then normalized. MEP amplitudes inferior to 0.1 mV were not considered. Z-scores were calculated using mean and standard deviations of each mini-block of 32 trials. Given the high variability of individual MEPs, Z-scores were used to increase the comparability of the mini-blocks, both within and between participants. Normalized MEP peak-to-peak amplitudes were subjected to a 2×2 repeated measures ANOVA with task (semantic vs. syllabic) and verb-category (action hand-action vs. non-hand action vs. non-action) as within-subject factors. All post-hoc comparisons between single factors were carried out using LSD Fisher's test (α ≤.05)

## Supporting Information

Appendix S1 Action-related and non-action related lexical items used in Experiments 1–3.
(0.09 MB DOC)
