# Supplementary material for: Effects of TMS on Different Stages of Motor and Non-Motor Verb Processing in the Primary Motor Cortex
Source: PLoS One. 2009 Feb 25;4(2):e4508. doi: 10.1371/journal.pone.0004508 (PMC2643000; doi:10.1371/journal.pone.0004508)
Supplement: Appendix S1 — Action-related and non-action related lexical items used in Experiments 1–3. (0.09 MB DOC) [file pone.0004508.s001.doc]

**Appendix S1**

Action-related and non-action related lexical items used in Experiments 1-3.

| *Hand-action verbs* |  | *Non-hand action verbs* |  |
| --- | --- | --- | --- |
| *abbottono, I button up* | *maneggio, I handle* | *accavallo, I cross* | *nuoto, I swim* |
| *abbraccio, I embrace* | *manipolo, I manipulate* | *accelero, I accelerate* | *oltrepasso, I outstrip* |
| *accarezzo, I caress* | *manometto, I tamper* | *addento, I bite* | *ondeggio, I stagger* |
| *acchiappo, I catch* | *martello, I hammer* | *ammicco, I wink* | *palleggio, I bounce* |
| *acconcio, I comb* | *mescolo, I steer* | *annuisco, I nod* | *passeggio, I stroll* |
| *afferro, I grasp* | *mitraglio, I shoot* | *annuso, I sniff* | *pedalo, I pedal* |
| *agguanto, I grab* | *mungo, I milk* | *arretro, I move back* | *percorro, I walk* |
| *allaccio, I fasten* | *palpeggio, I feel* | *avanzo, I move forward* | *peregrinare, I stroll* |
| *amalgamo, I mix* | *perquisisco, I frisk* | *ballo, I dance* | *pesto, I stamp on* |
| *ammanetto, I arrest* | *poto, I prune* | *bevo, I drink* | *piantono, I guard* |
| *annodo, I tie* | *prendo, I take* | *bighellono, I stroll about* | *piroetto, I twist* |
| *applaudo, I clap* | *pugnalo, I stab* | *calpesto, I stomp* | *proferisco, I utter* |
| *arrostisco, I roast* | *remo, I row* | *cammino, I walk* | *retrocedo, I demote* |
| *autografo, I sign* | *rimescolo, I reshuffle* | *cavalco, I ride* | *ridacchio, I chortle* |
| *avvito, I screw* | *riparo, I fix* | *circondo, I encircle* | *rido, I laugh* |
| *bastono, I thrash* | *saluto, I salute* | *corro, I run* | *rincorro, I run after* |
| *cesello, I carve* | *sbottono, I unbutton* | *danzo, I dance* | *salgo, I rise* |
| *clicco, I click* | *scaravento, I hurl* | *decellero, I slow down* | *saltello, I jig* |
| *coloro, I color* | *scavo, I dig* | *deglutisco, I swallow* | *salto, I jump* |
| *condisco, I dress* | *scrivo, I write* | *dondolo, I swing* | *sbuffo, I fume* |
| *cucio, I sew* | *sfoglio, I leaf through* | *espiro, I breathe out* | *scappo, I rush off* |
| *decoro, I decorate* | *sminuzzo, I chop* | *gareggio, I race* | *scavalco, I leap over* |
| *digito, I press* | *smonto, I dismantle* | *gattono, I crawl* | *scivolo, I slip* |
| *dipingo, I paint* | *soffriggo, I fry* | *gironzolo, I loiter* | *sgambetto, I trot* |
| *firmo, I sign* | *solletico, I tickle* | *girovago, I bum around* | *sgranocchio, I crunch* |
| *imbottisco, I pad* | *sparo, I shoot* | *imprigiono, I imprison* | *soffio, I blow* |
| *impugno, I clasp* | *spremo, I squeeze* | *inchino, I bow* | *sogghigno, I sneer* |
| *incateno, I chain* | *stappo, I uncork* | *indietreggio, I back off* | *striscio, I crawl* |
| *indico, I point* | *stiro, I iron* | *inseguo, I pursue* | *vagabondo, I rove* |
| *inscatolo, I pack in tins* | *stringo, I hold* | *marcio, I march* | *veleggio, I sail* |
| *intarsio, I sculpt* | *strofino, I rub* | *mastico, I chew* | *volteggio, I vault* |

| *Non-action Verbs* |  |  |  |
| --- | --- | --- | --- |
| *addoloro, I am distressed* | *elimino, I eliminate* | *manco, I miss* | *rinnego, I disown* |
| *adoro, I adore* | *eredito, I inherit* | *medito, I wonder* | *rinuncio, I give up* |
| *ambisco, I hanker* | *esagero, I exaggerate* | *memorizzo, I memorize* | *riprovo, I retry* |
| *analizzo, I analyze* | *esamino, I examine* | *miglioro, I improve* | *rispetto, I respect* |
| *angoscio, I am worried* | *esigo, I demand* | *moltiplico, I multiply* | *sbaglio, I mistake* |
| *annoio, I bore* | *esordisco, I debut* | *nego, I deny* | *sboccio, I blossom* |
| *annullo, I nullify* | *evito, I avoid* | *obbedisco, I obey* | *scado, I decrease in value* |
| *apprezzo, I appreciate* | *evolvo, I evolve* | *odio, I hate* | *scelgo, I choose* |
| *assumo, I assume* | *fallisco, I fail* | *offendo, I offend* | *scoccio, I annoy* |
| *attendo, I wait* | *favorisco, I promote* | *omologo, I approve* | *scommetto, I bet* |
| *auspico, I wish* | *formulo, I formulate* | *ostento, I boast* | *sconto, I discount* |
| *autorizzo, I authorize* | *fuorvio, I mislead* | *perdo, I lose* | *sconvolgo, I distress* |
| *bado, I take care of* | *garantisco, I guarantee* | *placo, I calm* | *soffro, I suffer* |
| *boccio, I flunk* | *gioisco, I rejoice* | *plagio, I crib* | *sogno, I dream* |
| *brillo, I shine* | *gradisco, I enjoy* | *poltrisco, I lounge* | *somiglio, I resemble* |
| *decido, I decide* | *gravo, I bear upon* | *possiedo, I own* | *sopporto, I tolerate* |
| *comincio, I begin* | *idolatro, I idolize* | *preferisco, I prefer* | *sospendo, I suspend* |
| *condivido, I share* | *ignoro, I ignore* | *preoccupo, I worry* | *stanzio, I allocate* |
| *condiziono, I influence* | *illudo, I deceive* | *prevedo, I predict* | *stimo, I estimate* |
| *conosco, I know* | *imparo, I learn* | *proibisco, I prohibit* | *stupisco, I astonish* |
| *consisto, I consist* | *influenzo, I influence* | *promuovo, I promote* | *sublimo, I subliminate* |
| *deludo, I disappoint* | *interesso, I interest* | *provo, I feel* | *subordino, I subdue* |
| *deplore, I deplore* | *interpreto, I interpret* | *provoco, I provoke* | *sudo, I sweat* |
| *deprimo, I depress* | *intristisco, I get sad* | *ragiono, I reason* | *sussisto, I subsist* |
| *desidero, I desire* | *invecchio, I grow old* | *rallegro, I am happy* | *taccio, I shut up* |
| *detesto, I detest* | *invidio, I envy* | *rappresento, I represent* | *tasso, I tax* |
| *dimentico, I forget* | *ipotizzo, I hypothesize* | *rendo, I convey* | *temo, I fear* |
| *distinguo, I distinguish* | *istruisco, I teach* | *resisto, I resist* | *tollero, I tolerate* |
| *divento, I become* | *lamento, I complain* | *ricevo, I receive* | *trascuro, I abandon* |
| *doto, I provide* | *legalizzo, I legalize* | *riconosco, I acknowledge* | *turbo, I trouble* |
| *dubito, I doubt* | *limito, I limit* | *rifletto, I think overt* | *vario, I change* |
| *elaboro, I elaborate* | *lucro, I earn* | *rimpiango, I regret* | *vinco, I win* |
